# Pleomorphic adenoma arising from the tail of the parotid gland – value of preoperative multi planar imaging: a case report

**DOI:** 10.1186/1757-1626-1-23

**Published:** 2008-07-03

**Authors:** R Vaidhyanath, S Harieaswar, C Kendall, P Conboy

**Affiliations:** 1 Department of Radiology, Leicester Royal Infirmary, University Hospitals of Leicester, Leicester, LE1 5WW, UK; 2 Department of Histopathology, Leicester Royal Infirmary, University Hospitals of Leicester, Leicester, LE1 5WW, UK; 3 Department of ENT, Leicester Royal Infirmary, University Hospitals of Leicester, Leicester, LE1 5WW, UK

## Abstract

**Background:**

Lesions of the 'tail' of the parotid gland are difficult to assess clinically and provide a diagnostic dilemma on imaging, especially in the axial plane. Pedunculated lesions of the 'tail' of parotid can be mistaken for an extra parotid lesion. Accurate localisation of these lesions on imaging is essential to assist the clinical diagnosis, to prevent inadequate/incomplete excision and complications, especially damage to facial nerve.

**Case Report:**

In this case report, we present a case of a pleomorphic adenoma arising from the 'tail' of the parotid gland, which on imaging, appears to be extra parotid in location. We also review the anatomy of the parotid 'tail' and relevant literature.

**Conclusion:**

Lesions of the parotid 'tail' are a diagnostic challenge to clinicians and Radiologists. Pedunculated lesions arising from the 'tail' of the parotid gland can appear extra parotid in location. Knowledge of parotid gland anatomy and use of multi planar imaging is essential in the accurate localisation of these lesions. This will also prevent inadequate or incomplete excision.

## Background

Lesions of the 'tail' of the parotid gland are difficult to assess clinically and provide a diagnostic dilemma on imaging, especially in the axial plane. Pedunculated lesions of the 'tail' of parotid can be mistaken for an extra parotid lesion. Accurate localisation of these lesions on imaging is essential to assist the clinical diagnosis, to prevent inadequate/incomplete excision and complications, especially damage to facial nerve.

In this case report, we present a case of a pleomorphic adenoma arising from the 'tail' of the parotid gland, which on imaging, appears to be extra parotid in location. We also review the anatomy of the parotid 'tail' and relevant literature.

## Case report

A 65 yr old female presented to the Ear Nose and Throat [ENT] clinic of our institution with a one-month history of a lump in the right neck. On examination, a mobile lump was palpable in the angle of the mandible/upper cervical region. A flexible endoscopy did not reveal any mucosal lesion in the posterior nasal space, oropharynx, hypo pharynx or in the larynx. Clinically a lymph node mass was considered as a possible diagnosis. Fine needle aspiration [FNA] and Magnetic Resonance Imaging [MRI] were then performed.

**Figure 1:**
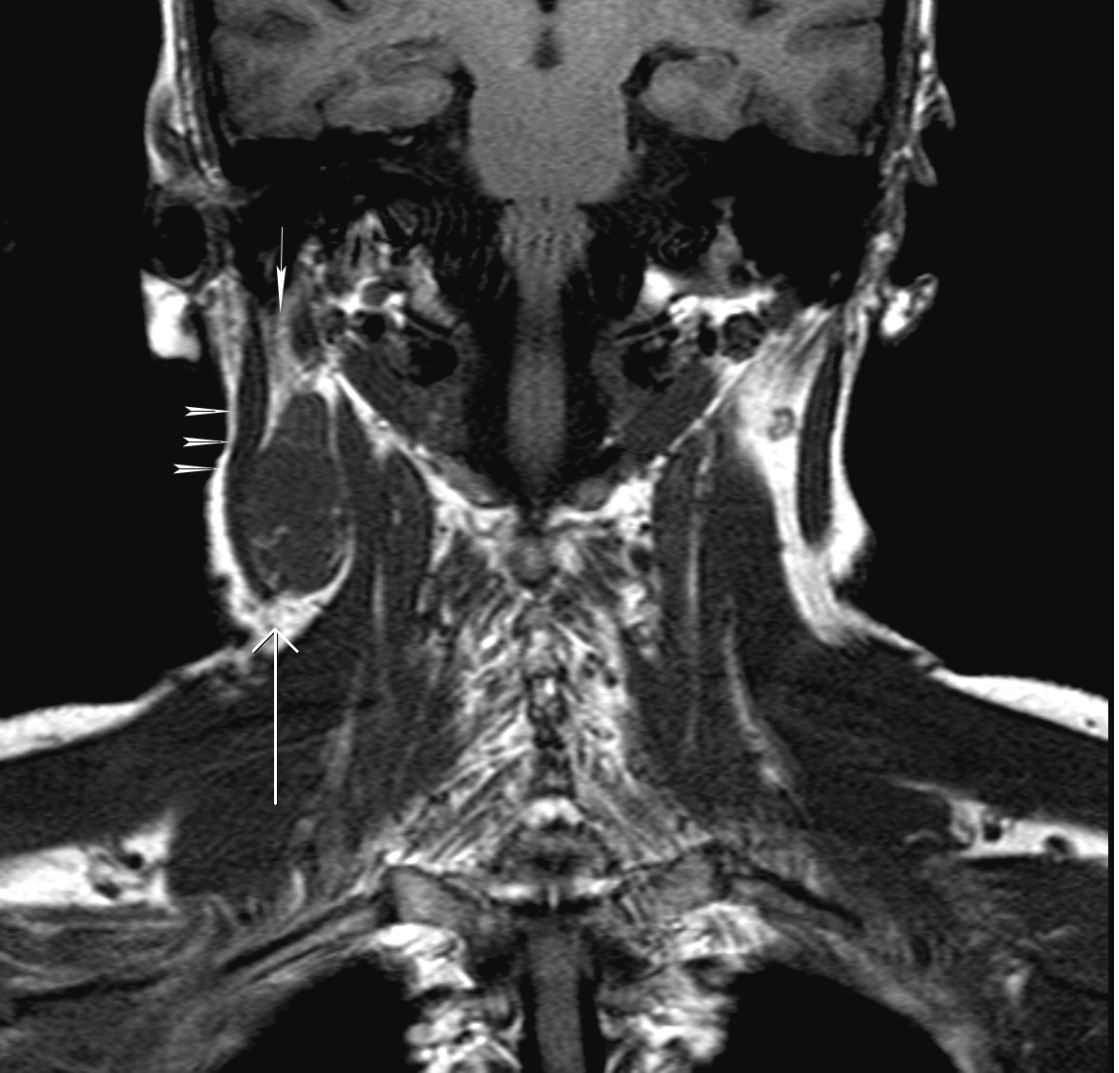
T1 Coronal image: There is a well defined mass (long white arrow) inferior to the right parotid gland (short white arrow) & medial to the sternocleidomastoid muscle (arrowheads).

**Figure 2:**
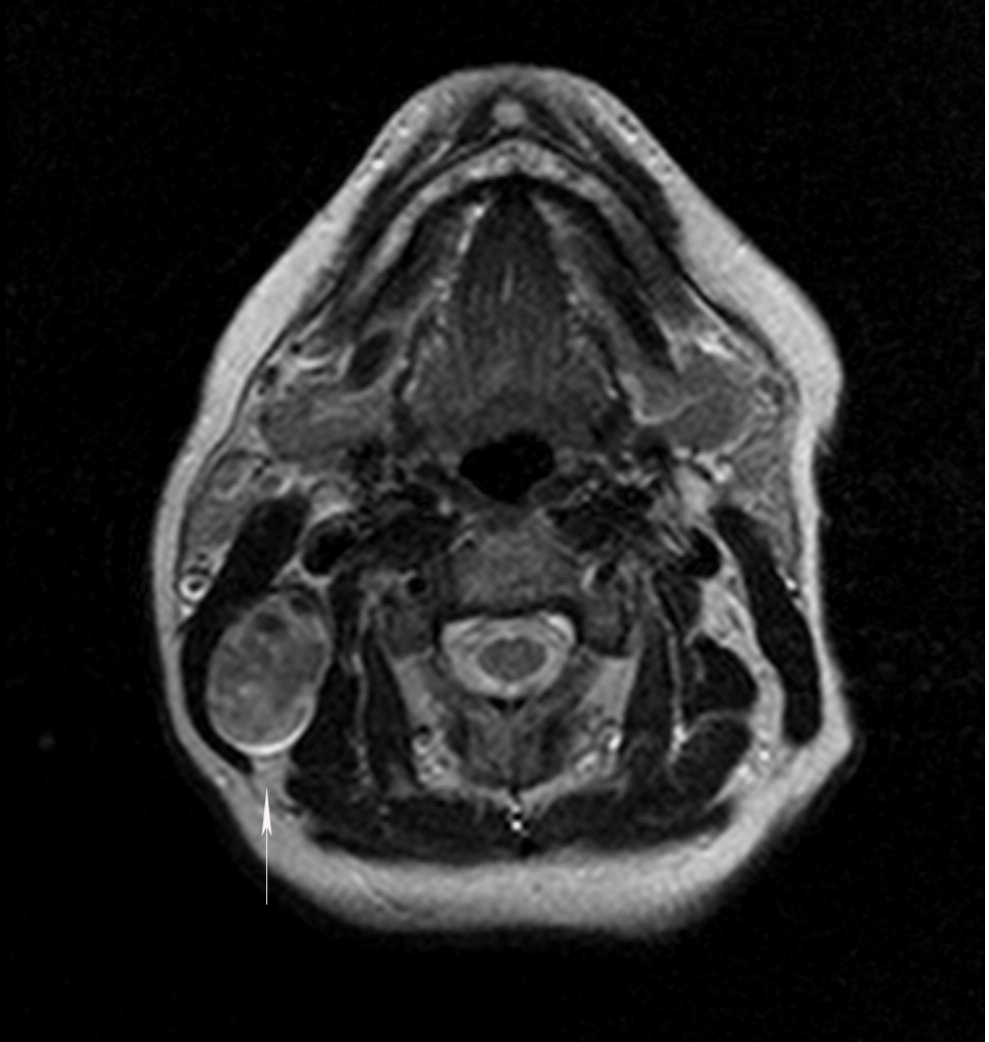
T2 Axial image: The lesion has mixed signal intensity. The lesion appears to be separate from the parotid gland.

**Figure 3:**
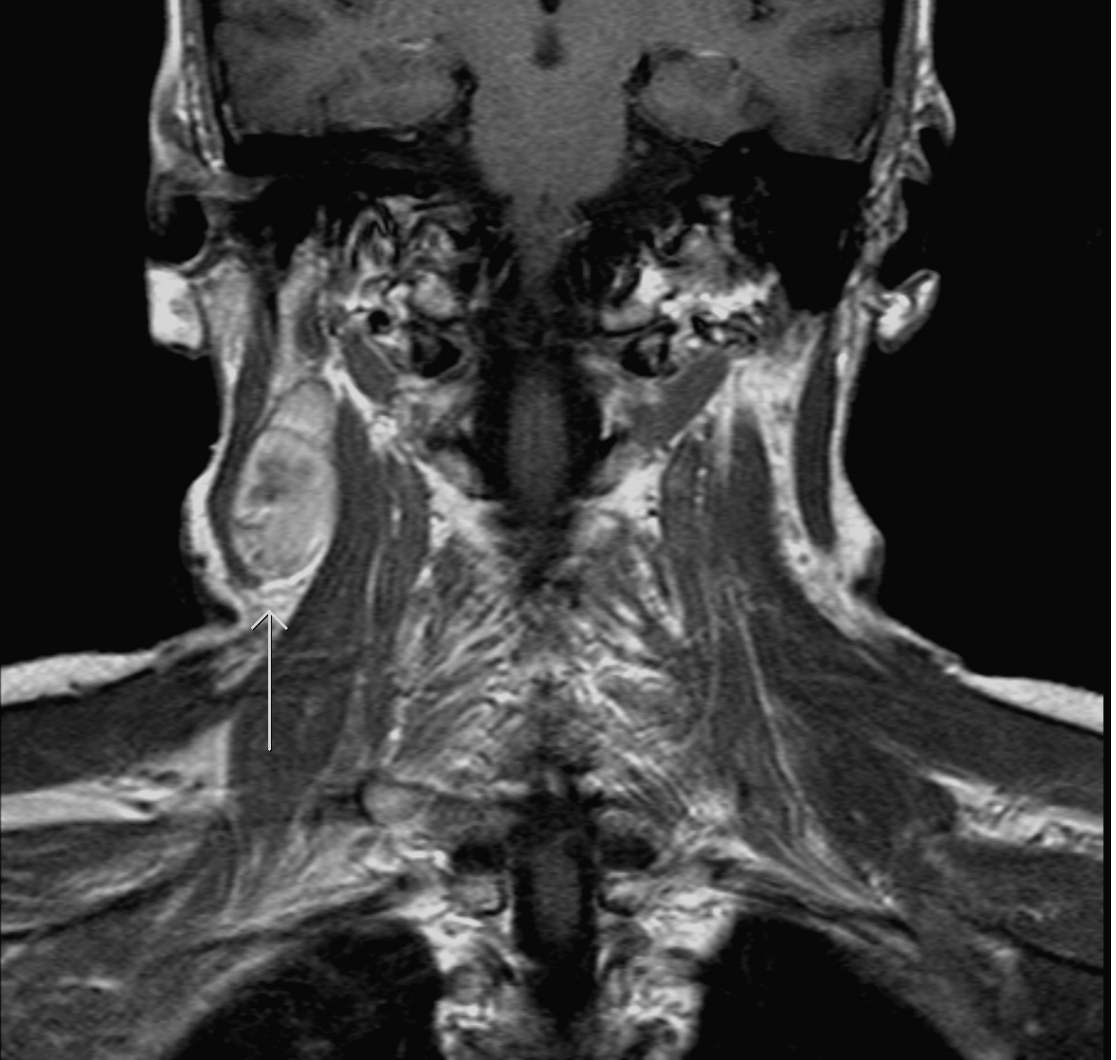
Post contrast T1 coronal image: There is moderate enhancement following contrast administration.

MRI showed a well-defined oval shaped mass measuring 3 × 2 cm just inferior to the right parotid gland. The epicenter of the lesion was in the inter muscular plane, medial to the sternocleidomastoid muscle but separate from it. It was heterogeneous on both T1 & T2 W images with a well defined margin (Fig 1 & 2). Following contrast administration, there was moderate heterogeneous enhancement (Fig 3) In view of the location of the lesion, a differential diagnosis of lymph nodal mass & Spinal Accessory nerve Schwannoma were considered.

FNA of the lesion showed a mix of bland epithelial cell, clusters of spindle cells and myxoid matrix, which was typical of a pleomorphic adenoma.

A decision to perform an excision biopsy was subsequently made. At surgery, the tumor was found to be a pedunculated mass arising from the inferior aspect of the tail of the parotid gland. It was closely related and superficial to the spinal accessory nerve but separate from it. The mass was excised completely incorporating a limited cuff of macroscopically normal parotid tissue, taking care not to injure the inferior branches of the facial nerve.

Histopathology of the excised specimen showed uniform proliferation of epithelial elements within a patially myxo-chondroid stroma, again, consistent with a diagnosis of a pleomorphic adenoma. There was no definite salivary gland tissue within the specimen.

## Discussion

The anatomy and description of the parotid 'tail' is confusing as it is not a distinct anatomical entity. It is a triangular shaped parotid tissue seen anterolateral to the sternocleidomastoid muscle, posterolateral to the posterior belly of diagastric muscle & deep to the platysma [1].

Hamilton et al [2] define the 'tail' of the parotid gland as inferior 2 cms of the superficial lobe of the gland. Many surgeons, however, consider the retromandibular portion of the superficial gland as the 'tail' of the parotid gland.

Lesions arising from the 'tail' of parotid gland can be challenging to the clinicians and is often mistaken as a nodal mass or a posterior submandibular space lesion. This is particularly true of a pedunculated mass arising from the inferior aspect of the parotid gland.

Imaging plays an important role, not in the diagnosis of the mass, but, to accurately locate the intra or extra parotid location of these lesions. This has important implications on the surgical management of the patient. Parotidectomy with facial nerve preservation is the method of choice in treating benign lesions of the parotid gland [3]. This technique is not only diagnostic but minimises the risk of recurrence in most cases, with a very low risk of permanent facial nerve injury. There is a significant risk of tumour recurrence, often multi focal, with incomplete excision [4].

On CT or MRI lesions arising from the parotid 'tail' could appear to be extra parotid in location especially in the axial plane. Lack of surrounding parotid tissue can only add to the confusion. Coronal images are helpful in assessing the location of the lesion. In general, lesions arising from the parotid 'tail' (intra parotid mass) are anterolateral to the sternocleidomastoid muscle while extra parotid masses are anteromedial to sternocleidomastoid muscle [2]. In our patient, the mass was seen anteromedial to the sternocleidomastoid muscle in the axial plane.

The standard approach to a neck mass is clinical examination followed by fine needle aspiration. An ultrasound examination combined with fine needle aspiration is the preferred choice. This allows the correct location of the lesion and ensures adequate material is obtained for cytological examination.

Lesions of the parotid 'tail' are a diagnostic challenge to the imager. Knowledge of the parotid gland anatomy & use of multi planar imaging is essential in the accurate localisation of these lesions.

## Conclusion

Lesions of the parotid 'tail' are a diagnostic challenge to clinicians and radiologists. Pedunculated lesions arising from the 'tail' of the parotid gland can appear extra parotid in location. Knowledge of parotid gland anatomy and use of multi planar imaging is essential in the accurate localisation of these lesions. This will also prevent inadequate or incomplete excision.

## Competing interests

The authors declare that they have no competing interests.

## Authors' contributions

RV, PC conceived the case report. SH prepared the manuscript. RV, PC, CK finalised the manuscript. All authors read and approved the final manuscript.

## Consent

Written informed consent was obtained from the patient for publication of this case report and any accompanying images. A copy of the written consent is available for review by the Editor-in-Chief of this journal.
